# Sociability Under Anthropogenic Change: From Individual Behaviour to Population‐Level Consequences

**DOI:** 10.1002/ece3.74205

**Published:** 2026-09-30

**Authors:** Paulina Kowalczyk, Barbara Pietrzak

**Affiliations:** ^1^ Institute of Ecology, Faculty of Biology University of Warsaw Warszawa Poland

**Keywords:** animal personality, anthropogenic change, behavioural plasticity, population resilience, sociability, social behaviour

## Abstract

Animal responses to anthropogenic environmental change depend not only on individual phenotypes, but also on how individuals associate with one another. Sociability—the tendency to seek or tolerate association with conspecifics—links individual behaviour to social organisation and population processes. In this review, we synthesise empirical and theoretical work to examine sociability as a mechanistic axis connecting individual behaviour, social networks and population processes under anthropogenic change. We distinguish sociability from broader concepts such as sociality and social complexity, and integrate evidence across taxa and levels of biological organisation. We examine how sociability mediates animal responses to habitat modification, climate change, pollution, disturbance, provisioning and non‐random mortality, and how these responses scale from social interactions to networks and broader social organisation. Across systems, sociability can either buffer or amplify the effects of anthropogenic change depending on how environmental disturbance reshapes the costs and benefits of association. By altering energetic constraints, predation risk, disease exposure, and access to information and resources, anthropogenic change can shift when, how, and for whom social associations are beneficial. Social ties may reduce short‐term stress and uncertainty, but they may also intensify competition, pathogen transmission, or social destabilisation. These outcomes are strongly context dependent, varying with species ecology, social system organisation, temporal scale and broader disturbance regimes. By positioning sociability as an important pathway through which anthropogenic change can scale from individuals to populations, we identify priorities for future research, particularly long‐term individual‐based studies that disentangle plasticity and consistency in social behaviour, and test how anthropogenic change shapes selection on social traits.

## Graphical abstract

Our review synthesises empirical and theoretical research on how anthropogenic environmental change reshapes animal social behaviour from individuals to populations. We use sociability as an individual‐level lens, distinguishing context‐dependent expression from repeatable interindividual differences and linking both to social relationships, network structure, population processes, and adaptive potential. We consider habitat modification, climate change, pollution, human disturbance, provisioning and non‐random mortality. We conclude that anthropogenic change reshapes the costs and benefits of social association through recurrent pathways, while outcomes depend on species ecology, social organisation, individual phenotype and social role, and the duration and predictability of disturbance.

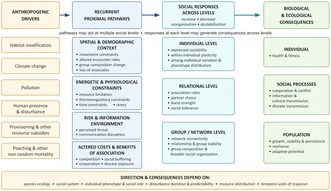

## 1 Introduction

The ongoing expansion of the human population has resulted in profound changes across almost all of Earth's ecosystems, often at a scale that remains underestimated (Pärtel et al. 2025). Behavioural change is among the earliest and most flexible responses of animals to environmental alteration (Wolf and Weissing 2012; Wong and Candolin 2015). However, animals respond to environmental change not only as solitary individuals interacting with physical conditions, but also as members of social environments shaped by repeated interactions with conspecifics.

A wide diversity of animal social systems has been described, yet how species are classified as social or solitary often depends on the criteria applied, leading to inconsistent and sometimes incompatible classifications across studies (Makuya and Schradin 2024). Importantly, even species commonly labelled as solitary typically engage in repeated, non‐random interactions with conspecifics, generating social environments that influence individual behaviour and fitness.

One important source of variation within social environments is interindividual differences in the tendency to associate with conspecifics in non‐aggressive contexts, commonly referred to as sociability (Réale et al. 2007). Here, we use sociability as an individual‐level perspective that complements, rather than replaces, descriptions of social organisation. This perspective is relevant not only to animals living in stable groups but also to species whose social relationships are temporary, fluid, or weakly structured. In group‐living species, variation in sociability among group members may be particularly important, as individuals with different social tendencies may fulfil different roles and contribute differently to group functioning. Moreover, sociability need not constitute a single, unitary trait but may be expressed through partly distinct behavioural facets, including spatial proximity, affiliative interactions, and association preferences. Although such interindividual variation has been documented across a wide range of taxa, sociability remains one of the least studied and least consistently defined domains of animal personality, contributing to conceptual ambiguity in the literature (Gartland et al. 2022; Martin, Jaeggi, and Koski 2023).

Throughout this review, we distinguish the context‐dependent expression of sociability from repeatable among‐individual differences in this tendency, which constitute a personality trait. Such consistency over time or across contexts does not imply fixed behaviour. Individuals may alter their expressed sociability in response to environmental conditions while repeatable differences among them persist. They may also differ in the magnitude or direction of this plasticity, as represented by individual behavioural reaction norms (Dingemanse et al. 2010; Mathot and Dingemanse 2014). Anthropogenic change may thus affect sociability through reversible within‐individual plasticity, developmental changes in social tendencies, or selective and demographic processes that alter the distribution of sociability phenotypes within populations. We use personality‐related terminology only where studies demonstrate repeatable among‐individual variation. Elsewhere, we refer to expressed sociability or social behaviour.

Anthropogenic environmental changes, including climate change, habitat fragmentation, pollution and direct interactions with humans, have been shown to affect animal social behaviour across taxa. These responses are often complex and context‐dependent, and may differ among facets of social behaviour. The diversity of metrics used to quantify sociability further complicates comparisons across studies and may contribute to apparently contradictory findings. Nonetheless, variation in sociability has been identified as an important determinant of fitness, while emergent social structures influence ecological processes such as information flow, disease transmission and population resilience.

In this review, we use sociability as an individual‐level lens for examining biological responses to anthropogenic change. Specifically, we ask how anthropogenic change affects the expression of different facets of sociability, how individuals differ in both their average social tendencies and behavioural plasticity, and how these individual‐level responses feed back to reshape social environments, population dynamics, and adaptive potential. To address these aims, we synthesise evidence across taxa and levels of biological organisation using a conceptual, integrative perspective. We first clarify terminology and conceptual frameworks surrounding sociality and sociability, then review behavioural responses to anthropogenic change, and finally discuss the implications of variation in sociability for population resilience and conservation.

### 1.1 Approach to Literature Synthesis

This review adopts a structured narrative approach to synthesise empirical and theoretical work on animal sociability in the context of anthropogenic environmental change. Rather than aiming to provide an exhaustive catalogue of studies or a quantitative meta‐analytic assessment, our goal is conceptual integration: to clarify terminology, identify recurrent mechanisms and link individual‐level variation in sociability with population‐ and group‐level processes relevant to adaptation and resilience in human‐altered environments.

Literature was identified through targeted searches in Web of Science and Google Scholar using combinations of keywords including ‘sociability’, ‘sociality’, ‘animal personality’, ‘social behaviour’, ‘social networks’, ‘anthropogenic change’, ‘climate change’ and related terms. These searches were complemented by backward and forward citation tracking of influential synthesis papers and highly cited empirical studies, as well as by the inclusion of recent work that illustrates emerging approaches or underrepresented taxa.

Given the pronounced heterogeneity of taxa, study systems and methodological approaches used to investigate social behaviour, a formal systematic review or meta‐analysis was not considered appropriate for the aims of this synthesis. Measures of sociability vary widely across studies, ranging from proximity‐based metrics and affiliative interactions to social network position, group‐level descriptors and culturally mediated behaviours. This diversity limits direct quantitative comparison and effect size estimation. Consequently, this review focuses on identifying patterns, trade‐offs and feedbacks that recur across systems rather than on aggregating outcomes statistically.

The synthesis is explicitly multi‐level. We integrate evidence spanning individual behavioural tendencies, dyadic relationships, social networks and population‐level outcomes, with particular attention to how anthropogenic drivers may differentially affect these levels. By distinguishing individual‐level sociability from sociality as a broader property of social systems, the review aims to provide a coherent framework for interpreting seemingly contradictory findings across the literature.

We acknowledge that the available literature is unevenly distributed across taxa, with a strong representation of mammals and primates. Rather than attempting artificial taxonomic balance, we treat this bias as an informative feature of the field and highlight non‐mammalian examples where they illuminate general mechanisms. Identifying such gaps is an explicit aim of this synthesis and a basis for outlining future research directions.

## 2 Sociality, Sociability and Social Complexity

### 2.1 Locating Sociability Within Broader Social Systems

Alongside growing recognition of anthropogenic environmental change (Barnosky et al. 2011; Neukom et al. 2019), behavioural ecology has undergone a conceptual shift towards greater attention to individuals and interindividual variation. This shift has brought renewed focus on traits related to social behaviour, including sociability, understood here as the individual tendency to associate with conspecifics. Given this growing interest, clarifying the terminology used to describe animal social behaviour is a necessary first step, as imprecise or inconsistent usage can obscure rather than advance understanding.

To situate this individual‐level lens within broader social systems, we draw on Hinde's (1976) framework, in which repeated social interactions give rise to social relationships and, in turn, social structure, and on Kappeler's (2019) distinction among social organisation, social structure, mating system and care system. These frameworks help locate individual social tendencies within the wider social contexts that individuals both shape and experience, while also highlighting the persistent ambiguity of many ‘social’ terms.

Importantly, classification of species as ‘solitary’ or ‘social’, typically based on social organisation (i.e., social unit size and composition), does not necessarily reflect the extent or structure of social interactions among individuals. Species labelled as solitary can engage in repeated, non‐random interactions that contribute to complex social structures (Kappeler 2019; Makuya and Schradin 2024; Heeres et al. 2024). Additionally, the components of the social system used by authors to determine species' sociality differ. As a result, estimates of how many mammalian species are solitary vary widely across studies, ranging from over two‐thirds to less than a quarter, depending on the criteria applied (Lukas and Clutton‐Brock 2013; Smith et al. 2017; Kalbitzer et al. 2017; Makuya and Schradin 2024). Species‐level labels are therefore an uncertain guide to the range of individual social tendencies expressed within a species.

Definitions of ‘sociality’ vary substantially across the literature. Some authors define sociality primarily in terms of group living, describing it as the degree to which individuals live with conspecifics in groups or societies (Eisenberg 1966; Smith et al. 2017). This perspective is echoed in influential syntheses that equate being social with group living (Ward and Webster 2016). However, such definitions largely emphasise social organisation while downplaying other components of social systems, contributing to ongoing ambiguity in how sociality is assessed and compared across studies (Kappeler 2019; Makuya and Schradin 2024). They also capture a different level of social variation from the individual‐level tendencies considered here.

At the individual level, behavioural ecologists have increasingly focused on consistent interindividual differences in behaviour, commonly referred to as animal personalities (Sih et al. 2004; Réale et al. 2007). Within this framework, sociability is typically defined as an individual's tendency to associate with conspecifics in non‐aggressive contexts. Although this trait has been documented across a wide range of taxa, its conceptualisation remains inconsistent, with sociability and sociality often used interchangeably to describe individual‐level variation (Réale et al. 2007; Gartland et al. 2022).

This inconsistency is particularly evident when individual differences are described using terminology traditionally applied to species‐level or group‐level phenomena. While sociability refers to interindividual variation in social tendencies, the term ‘sociality’ has historically been used to characterise group‐living species. Applying the latter to individual‐level traits can therefore be misleading, especially given that variation in sociability also occurs in species not considered socially organised, such as semi‐solitary orangutans (*Pongo* spp.; Schuppli et al. 2020; Mörchen et al. 2024). Interindividual differences have been documented not only in the general propensity to associate with conspecifics—labelled as sociability or, in some studies, as sociality—but also in more context‐specific social behaviours. These include variation in dominance and leadership styles, cooperation, social bonding, conflict resolution, social learning, affiliation, and play, among others (Martin, Jaeggi, and Koski 2023). Together, these findings indicate that individual variation in the social domain extends beyond a single behavioural axis.

Further ambiguity arises from attempts to distinguish sociability from other behavioural axes, particularly aggression. While some definitions explicitly exclude interactions driven by aggression (Gartland et al. 2022), affiliative and aggressive behaviours are often intertwined within social systems and jointly contribute to social stability (Martin, Jaeggi, and Koski 2023). For example, in ring‐tailed lemurs ( *Lemur catta* ), individuals that occupy central positions in affiliative networks may also be frequent initiators of aggression, with social network centrality remaining consistent across multiple interaction types (Kulahci et al. 2018).

Reflecting this complexity, some authors have proposed explicitly multidimensional approaches to sociability. For example, Menz et al. (2020) distinguish between ‘gregariousness’, defined as an individual's propensity to be near others regardless of their identity, and ‘differentiated social relationships’, referring to an individual's direct and indirect relationships with particular conspecifics. These components capture distinct aspects of social behaviour and require different methodological approaches for quantification, as discussed below.

These inconsistencies complicate cross‐study comparisons and obscure links between individual behaviour and social systems. As Hinde (1976) emphasised, progress in understanding social behaviour requires coherent conceptual frameworks. Clarifying how sociality and sociability are defined and applied is therefore a prerequisite for integrating insights from animal personality research into broader studies of social complexity and evolution.

In line with this broader perspective, we use sociability throughout this review to refer to individual‐level tendencies to seek or tolerate non‐aggressive association with conspecifics. We examine both their context‐dependent expression and interindividual variation, and how these contribute to social relationships, networks, and broader social organisation (see also van Leeuwen et al. 2024).

### 2.2 Sociability Assessment and Different Views on Personality

Among the five commonly differentiated axes of animal personality—boldness, activity, exploration, sociability and aggressiveness (symbolically illustrated in figure 1 in Pietrzak 2025)—sociability remains one of the least studied (Gartland et al. 2022). Owing to its inherently social nature, interindividual differences in sociability are not always examined within a personality framework, that is, as behavioural differences consistent across time and contexts (Réale et al. 2007), nor are they consistently labelled as such.

A wide range of metrics has been used to assess sociability, including time spent in proximity to conspecifics, propensity to engage in affiliative interactions, or participation in species‐typical social behaviours. Importantly, the choice of metric is often shaped by species ecology, with, for example, shoaling tendencies commonly used in fish and grooming behaviour frequently employed in primates.

In recent years, social network analysis has become an increasingly popular approach for assessing interindividual differences in sociability and broader patterns of social behaviour (Vital and Martins 2009; Gartland et al. 2022; Blumstein et al. 2023). By quantifying patterns of interactions among individuals within a population, this approach enables the study of social structure and facilitates comparisons across taxa, albeit with certain limitations (Castles et al. 2014). Importantly, social network analysis allows individual‐level interactions to be interpreted in the context of population‐wide social structure (Krause et al. 2010).

When repeatable across time and contexts, an individual's position within a social network can itself be considered a personality trait or used as a proxy for sociability (Krause et al. 2010; Aplin et al. 2015). Social network metrics differ in the aspects of social behaviour they capture, ranging from the number and frequency of interactions to measures that account for the social importance of specific connections (Kulahci et al. 2018). While this diversity allows for nuanced insights into social behaviour, it also requires careful interpretation, as different metrics may reflect distinct social traits rather than a single dimension of sociability (Vital and Martins 2009).

A further challenge in assessing sociability arises from reliance on single behavioural measures that may fail to capture its multidimensional and context‐dependent nature (Martin, Jaeggi, and Koski 2023). As emphasised by Martin, Jaeggi, and Koski (2023), personality can be conceptualised either as a repeatable behavioural outcome emerging from interactions between individuals and their social environments, or as a set of underlying mechanisms that constrain behaviour across contexts. Importantly, behavioural consistency does not imply fixed trait values, as individual tendencies may shift with age or environmental conditions, particularly across artificial experimental settings and natural social environments.

The social context in which behaviour is expressed can further influence the assessment of sociability. Although many studies attempt to control for social effects by testing individuals in isolation, trait expression may differ substantially between social and asocial contexts (Magnhagen and Bunnefeld 2009). For instance, in sticklebacks ( *Gasterosteus aculeatus* ), correlations between activity and competitiveness depend on group size (Webster et al. 2007), while in European perch ( *Perca fluviatilis* ), behavioural tendencies differ between solitary and group settings but remain predictably ordered among individuals (Magnhagen and Bunnefeld 2009). These findings suggest that isolating individuals may not always yield ecologically valid measures of sociability, particularly for species that routinely engage in social interactions (Martin, Jaeggi, and Koski 2023).

### 2.3 From Individuals to Populations and Back

As individuals constitute the building blocks of populations and are the entities that directly interact, the content, quality and patterning of social relationships emerging from repeated interactions give rise to social structures shaped by individuals' decisions about with whom and how often to associate (Kappeler 2019; Cantor et al. 2021). Both individuals and the societies they form are dynamic. Group size, composition, and patterns of interaction change over time, while individuals acquire social competence as they mature or learn from conspecifics (Cantor et al. 2021). To fully understand the ecological and evolutionary consequences of variation in social behaviour, it is therefore essential to consider the reciprocal links between individual‐level traits and population‐level social organisation.

Social interactions, relationships, and resulting social structures are strongly influenced by the characteristics of individual group members (Figure 1). For example, in bonobos ( *Pan paniscus* ), individuals with similar levels of sociability derive greater benefits from social bonds with one another than from bonds with less similar conspecifics, indicating assortative patterns of social affiliation (Verspeek et al. 2019).

**FIGURE 1:**
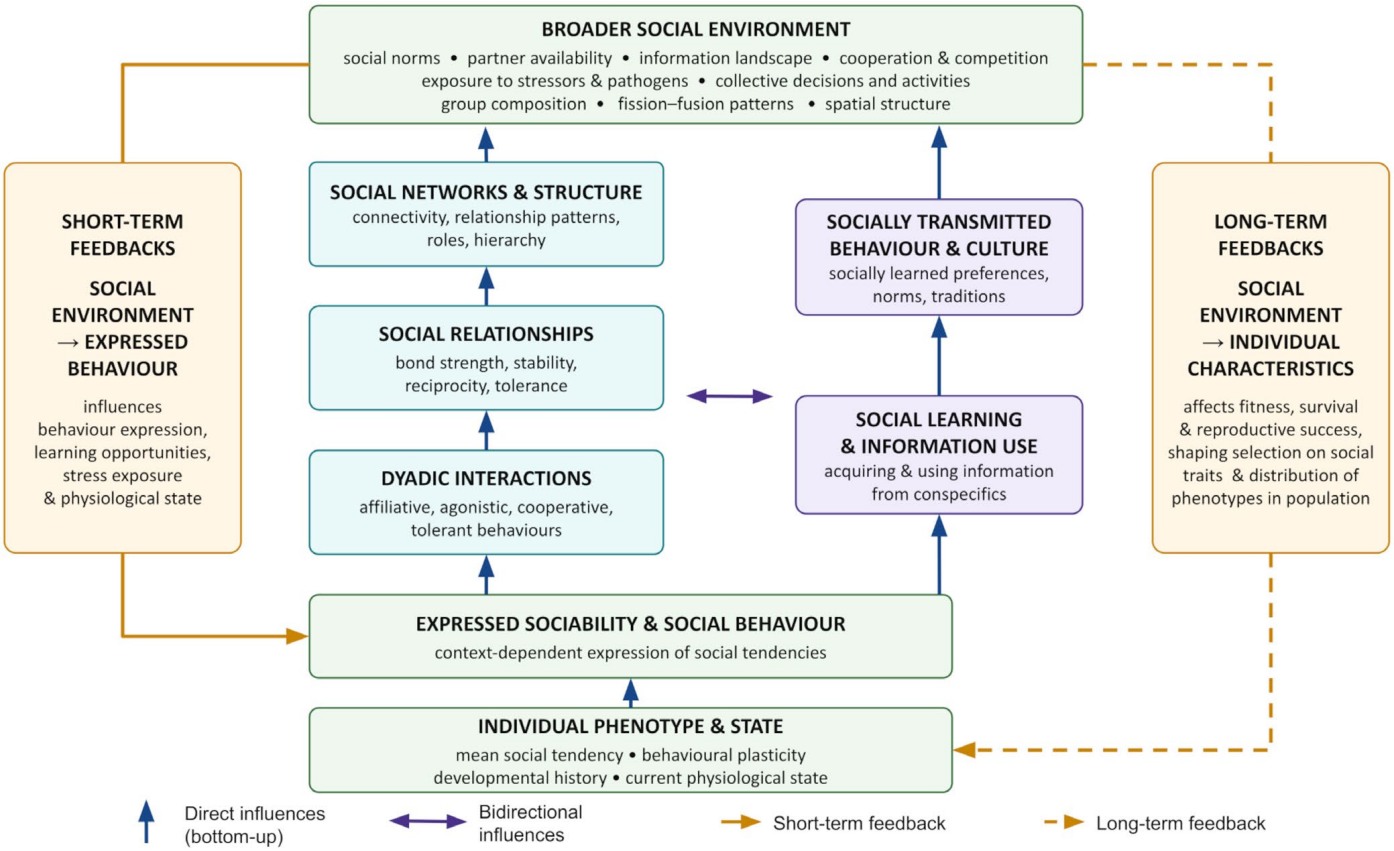
Conceptual framework linking individual social phenotypes with broader social systems and feedbacks across levels of social organisation. Bottom‐up processes connect expressed sociability and social behaviour with interactions, relationships, networks, social learning and culture, while the broader social environment feeds back on individuals over short and longer timescales. Not all interactions shown constitute sociability per se; some represent broader social processes that shape its expression and consequences. Social learning is shown separately, although it is embedded in interactions, relationships and network structure. The broader social environment is partly emergent from interactions and relationships and partly shaped by demographic and spatial organisation.

A particularly well‐known illustration of how individual traits can reshape group‐level social dynamics comes from a population of olive baboons ( *Papio anubis* ). Following a tuberculosis outbreak that disproportionately removed more aggressive males, the remaining group exhibited elevated levels of grooming and proximity, resulting in a markedly more tolerant social environment. Notably, this ‘pacific culture’ persisted even after all original males had been replaced by immigrants, highlighting how individual‐level selection can generate enduring changes in social structure (Sapolsky and Share 2004).

Such disproportionate effects of particular individuals on group processes have led to the concept of ‘keystone’ individuals or ‘gatekeepers’ or ‘brokers’—group members whose behaviour strongly influences social dynamics, including information flow and conflict regulation (Vital and Martins 2009). In pig‐tailed macaques ( *Macaca nemestrina* ), experimental removal of a small subset of individuals performing policing roles resulted in smaller, less diverse and less integrated social networks, demonstrating how the behaviour of particular individuals can stabilise group‐level social structure (Flack et al. 2006).

Social composition and broader group‐level characteristics can, in turn, shape the expression of behaviour in individuals. In African elephants ( *Loxodonta africana* ), the presence of younger males and a dominant, socially integrated ‘keystone’ male was associated with changes in the affiliative, aggressive and anxiety‐related behaviour of adult males, illustrating how local social composition can shape individual behavioural expression (O'Connell‐Rodwell et al. 2024). In vervet monkeys ( *Chlorocebus pygerythrus* ), stable between‐group differences in sociability have been documented that cannot be fully explained by ecological or demographic variables. Dispersing males adjust their sociability levels to match those of the groups they join, suggesting that group‐level social environments can shape individual behavioural tendencies (Kerjean et al. 2024).

Similar socially mediated effects on personality expression have been observed in other taxa. In the social spider *Stegodyphus dumicola* , interactions with conspecifics influence individual boldness, with less‐bold individuals increasing their boldness when embedded in bolder groups (Hunt et al. 2018). Likewise, common marmosets ( *Callithrix jacchus* ) exhibit group‐level similarity in personality traits, a pattern thought to be adaptive in highly cooperative species (Koski and Burkart 2015).

Two non‐mutually exclusive hypotheses have been proposed to explain such patterns. The conformity hypothesis suggests that individuals converge behaviourally towards group norms, whereas the social niche specialisation hypothesis posits that competition for social roles drives individuals to diverge from one another, thereby deepening interindividual differences (Bergmüller and Taborsky 2010; McCune et al. 2018). Despite their differences, both frameworks emphasise the central role of social context in shaping individual behaviour.

Individual sociability unfolds over lifetime and social experiences during early development can have lasting effects on individual social competence and behavioural strategies. In female rhesus macaques ( *Macaca mulatta* ), individuals reared with limited social exposure exhibit higher rates of stereotypic and submissive behaviours and reduced social competence later in life compared with socially reared peers (Kempes et al. 2008, 2009).

Comparable long‐term consequences of the social environment have been documented in other taxa. Group‐housed male zebra finches ( *Taeniopygia guttata* ) show enhanced social interaction rates, accelerated song and plumage development, and increased courtship and aggression relative to pair‐housed individuals, indicating that social exposure influences both behavioural and morphological maturation (Bölting and von Engelhardt 2017).

Importantly, developmental outcomes depend not only on the amount of social exposure but also on the quality and composition of social partners. Studies in cowbirds ( *Molothrus ater* ) and guppies (*Poecilia* spp.) demonstrate that access to socially competent or demographically biased groups can alter communication patterns and mating strategies, highlighting the role of social environment in shaping individual behavioural trajectories (White et al. 2007; Řežucha and Reichard 2014).

The reciprocal relationship between individual sociability and the social environment is consequential because associations with conspecifics entail both benefits and costs. Social interactions can provide access to information, enhance predator detection, facilitate cooperation, and support social learning, but they may also increase competition and exposure to pathogens (Alexander 1974; Pilakouta et al. 2023). As environmental conditions change, individuals must therefore continuously balance these trade‐offs to navigate their social and ecological landscapes.

Affiliative social interactions can facilitate cooperative behaviours by reducing social tension and stabilising relationships within groups. In adult chimpanzees ( *Pan troglodytes* ), social play increases during periods of heightened mate competition and occurs more frequently between individuals with a recent history of aggression, suggesting that play functions to mitigate conflict and maintain cooperation (Samuni et al. 2024). Individuals that have previously engaged in play are also more likely to cooperate during hunts and intergroup encounters, highlighting the role of affiliative interactions in enabling coordinated action (Samuni et al. 2024).

Familiarity among interaction partners can further reduce the costs of social living. Female eastern grey kangaroos ( *Macropus giganteus* ) have preferred and avoided social associates and spend more time foraging and less time on vigilance when in the presence of preferred partners, indicating that stable social relationships can enhance foraging efficiency (Carter et al. 2009).

Beyond facilitating cooperation, social relationships shape the flow of information within populations. Social learning allows individuals to acquire experience‐based knowledge from conspecifics and can provide a flexible mechanism for adapting to novel or changing environments (Allen et al. 2013). However, individuals differ in how and when they use socially acquired information. In an experiment with wild‐caught great tits ( *Parus major* ), birds assigned as immigrants relied most strongly on payoff‐biased social learning when both habitat cues and foraging payoffs changed but relied more on individual learning when either remained unchanged, whereas experimental residents largely retained their foraging strategies (Chimento et al. 2024).

The availability of social learning opportunities is itself shaped by social structure and individual sociability. In migrant unflanged male orangutans, increased food availability leads to longer association times and greater social tolerance, which in turn increases rates of observational learning. Moreover, individuals originating from more sociable populations show higher peering rates, suggesting that both ecological conditions and the social environment of their population of origin influence access to social information (Mörchen et al. 2024). Together, these findings illustrate how affiliative interactions and social structure jointly determine the informational landscape of populations.

While social interactions facilitate cooperation and information transfer, they also create pathways for the transmission of diseases and parasites. Consequently, the same social networks that confer benefits can impose substantial costs. In giraffes ( *Giraffa camelopardalis* ), transmission networks of *Escherichia coli* more closely resemble social than spatial networks, and individuals with higher numbers of social connections are also more highly connected within pathogen transmission networks (VanderWaal et al. 2014).

Highly connected individuals are therefore often considered at greater risk of infection and are frequently discussed as potential ‘super‐spreaders’ (Hamede et al. 2009). However, the relationship between sociability, network position, and disease dynamics is not straightforward. Social buffering—the health benefits derived from social integration—may reduce susceptibility to infection, complicating predictions about disease outcomes.

Empirical evidence highlights this complexity. In Grant's gazelles ( *Nanger granti* ), highly connected individuals show increased infection with some parasites (coccidia) but reduced burdens of others (lungworms), indicating that different pathogens respond differently to host social behaviour (Williams et al. 2017). These findings illustrate that the epidemiological consequences of sociability depend not only on social network structure but also on pathogen traits and host–parasite interactions.

As a result, variation in sociability does not translate uniformly into disease risk. Whether individuals become super‐spreaders, buffered against infection, or experience mixed outcomes depends on the interaction between individual behavioural tendencies and the social structures they co‐create.

Interindividual differences in sociability can have contrasting fitness consequences and may therefore be subject to natural selection (Martin, Jaeggi, and Koski 2023). Higher sociability has been associated with increased survival, improved health and greater reproductive success in some systems (e.g., Mcdonald 2007; Silk et al. 2009; Vander Wal et al. 2015; Turner et al. 2021), but such relationships are not universal. In eastern grey kangaroos, more sociable females exhibit reduced reproductive success (Menz et al. 2020), while in crested macaques ( *Macaca nigra* ) no clear association between grooming rates and birth probability has been detected (Duboscq et al. 2023).

The fitness consequences of sociability are particularly challenging to interpret because they emerge from interactions between individual traits and group‐level social environments. In mountain gorillas ( *Gorilla beringei beringei* ), the effects of strong social bonds on female health and reproduction differ between small and large groups, illustrating how group context can reverse the direction of fitness associations (Morrison et al. 2025). Moreover, different measures of sociability capture distinct aspects of social behaviour. For example, in giraffes, gregariousness better predicts female mortality risk than bond strength or network integration, underscoring the importance of matching behavioural metrics to biological questions (Bond et al. 2021).

Together, these findings demonstrate that sociability is neither universally beneficial nor universally costly. Instead, its fitness consequences emerge from interactions between individual behavioural tendencies, the social structures they help generate, and the ecological contexts that shape both. Recognising these reciprocal links across levels is essential for understanding how variation in sociability shapes social evolution and population responses to environmental change, particularly under increasing anthropogenic pressures.

## 3 Behavioural Response to Anthropogenic Change

Human activities have become a dominant force shaping environmental conditions across the globe, exposing organisms to rapid and often unprecedented ecological change (Barnosky et al. 2011; Neukom et al. 2019). Understanding how organisms respond to these changes is of critical importance given accelerating biodiversity loss, including rising rates of species extinction (Komdeur and Ma 2021; Cowie et al. 2022).

Behaviour plays a central role in mediating interactions between organisms and their environments and is often among the first observable responses to environmental change (Sih et al. 2011; Wolf and Weissing 2012). Human‐induced rapid environmental changes (HIRECs) have been shown to elicit strong behavioural responses across taxa (Gunn et al. 2022), with consequences for population persistence and stability. As such, behavioural responses provide a key window into how species cope with altered ecological conditions (Testard et al. 2021).

Animals may respond to environmental change through dispersal, evolutionary adaptation or phenotypic plasticity (Wong and Candolin 2015). Each of these strategies, however, is constrained. Dispersal may be limited by habitat fragmentation, evolutionary change may be too slow to match the pace of current environmental shifts, and genetic variation—the raw material for evolution—is often reduced by anthropogenic population declines (Wolf and Weissing 2012; Fisher et al. 2021). Under such conditions, phenotypic plasticity may provide a particularly important mechanism for short‐term persistence.

Phenotypic plasticity, broadly defined as the capacity of a genotype to produce different phenotypes under varying environmental conditions (Thibert‐Plante and Hendry 2011), can involve developmental, epigenetic and behavioural change. Although plastic responses are often adaptive, they can also be maladaptive under certain conditions, highlighting the importance of the environmental contexts in determining their fitness consequences (Ghalambor et al. 2007).

Social processes can substantially expand the scope of behavioural plasticity. Individuals may adjust their behaviour through individual learning or by acquiring information from conspecifics via social learning, enabling rapid responses to changing environments (Cantor and Whitehead 2013; Whiten 2021; Fox et al. 2024). Socially transmitted knowledge—such as information about foraging strategies, migratory routes, or novel resource use—can facilitate behavioural flexibility and the spread of innovations (Brakes et al. 2021; Kalan and Luncz 2025). For example, in Indo‐Pacific bottlenose dolphins ( *Tursiops aduncus* ), tool‐using individuals experienced a smaller post‐heatwave decline in survival than non‐tool users, possibly because their specialised, socially transmitted, foraging behaviour provided access to a less severely affected foraging niche (Wild et al. 2019). Because social learning is embedded within social relationships and network structures, understanding responses to environmental change requires explicit consideration of social organisation and individual social tendencies.

Consistent behavioural differences among individuals do not imply behavioural rigidity, as personality and plasticity are not mutually exclusive (Mathot and Dingemanse 2014). Individuals can differ not only in their average behavioural tendencies but also in their degree of behavioural plasticity. For instance, in common bottlenose dolphins ( *Tursiops truncatus* ), individuals differed in their plastic responses of gregariousness to short‐term variation in food availability, revealing among‐individual variation in reaction norms despite stable population‐level social structure (Fisher and Cheney 2023). Such variation in behavioural plasticity may itself be heritable and subject to selection (Dingemanse et al. 2010).

Behavioural diversity within populations can have important fitness consequences and shape population‐level dynamics. In the social spider *Anelosimus studiosus* , individuals in colonies composed of contrasting behavioural types achieve higher fitness than individuals in more homogeneous groups (Pruitt and Riechert 2011). At the population level, this diversity can buffer against environmental variability through the portfolio effect and increase the likelihood that at least some individuals can cope with novel conditions, an insurance effect that may be critical under rapid anthropogenic change (Wolf and Weissing 2012). These considerations support the view that behaviour—alongside genetic and species diversity—constitutes an important component of biodiversity in its own right (Berger‐Tal et al. 2025).

Together, these findings demonstrate that population‐level responses to anthropogenic environmental change cannot be fully understood from mean trait values alone, but emerge from interindividual variation and context‐dependent behavioural plasticity. Evidence from aquatic systems further shows that such individual‐level differences can mediate how populations respond to anthropogenic pressures, highlighting the generality of individual‐based mechanisms across ecological contexts (Fudali and Pietrzak 2024). In the following section, we focus specifically on how anthropogenic environmental change alters the expression of sociability and broader patterns of social interaction, and how these responses feed back into individual fitness, population dynamics, and adaptive potential.

## 4 Sociability Under Anthropogenic Change

Social dynamics are inherently context‐dependent. To maximise the benefits of social living under changing environmental conditions, social interactions and group cohesion must be sufficiently flexible to track shifting ecological demands (Seebacher and Krause 2019). In habitats characterised by unpredictability or chronic stress—such as arid savannas, where many species already operate close to their physiological limits—sociality and cooperation may be particularly important for sustaining individual performance and population persistence (Komdeur and Ma 2021). By reducing individual costs and enhancing access to key resources such as food and predator information, social relationships can buffer individuals and groups against environmental challenges.

Environmental change can affect social behaviour at multiple levels. Because individual phenotypes shape social structure, and social interactions in turn influence individual phenotypes, changes in social dynamics often involve feedbacks between individuals and populations (Seebacher and Krause 2019). This bidirectional coupling is mediated by social plasticity—the capacity of individuals to adjust their behaviour in response to their social environment—which generates feedback between direct genetic effects and indirect genetic effects arising from interactions among social partners (Wolf et al. 1998; Martin, Jaeggi, and Koski 2023). Through these feedbacks, anthropogenic change may alter not only individual behaviour but also the structure and functioning of entire social systems.

Given this complexity, behavioural responses in the social domain are rarely uniform. Changes in social networks may be heterogeneous, with relationships changing in different ways, or homogeneous, with interaction strengths shifting proportionally while the overall pattern of the network's structure remains intact (Franz and Alberts 2015). Environmental change may also influence social behaviour directly, by altering individual decision‐making, or indirectly, by modifying group size and composition (Blumstein et al. 2023). Both pathways can have cascading consequences for individual fitness, population dynamics, and resilience. In interpreting the evidence reviewed below, we therefore distinguish among changes in expressed social behaviour, within‐individual plasticity in sociability, and shifts in the distribution of sociability phenotypes within populations. Because many studies do not assess behavioural repeatability across time or contexts, environmentally induced changes in social behaviour should not automatically be interpreted as changes in sociability as a personality trait.

Variation in sociability—the tendency of individuals to associate with conspecifics—plays a central role in shaping how populations respond to anthropogenic change. As discussed in the previous section, social learning and culture can facilitate rapid behavioural adjustments, enabling individuals to exploit novel resources or cope with altered conditions. Because opportunities for social learning depend on patterns of association and social tolerance, the sociability of individuals is likely to influence the breadth and complexity of cultural repertoires within populations they co‐create (Whiten and Van Schaik 2007; Bolcato and Aplin 2025). Comparative studies of great apes, for example, suggest that greater tolerance for proximity among conspecifics is associated with richer and more cognitively complex cultural traditions, highlighting sociability as a key mediator between social structure, learning opportunities, and adaptive potential (Whiten and Van Schaik 2007).

Together, these considerations suggest that anthropogenic environmental change can reshape expressed sociability, social interactions and broader social organisation through multiple, interacting pathways, with consequences that extend from individual behaviour to population‐level processes. In the following sections, we examine how indirect environmental changes—such as habitat modification, climate change and pollution—and direct human pressures influence expressed sociability and broader social dynamics across taxa, and discuss the implications of these effects for population resilience and conservation.

### 4.1 Indirect Consequences of Human Activity

Indirect anthropogenic effects influence animal sociability not through direct interactions with humans, but by reshaping the ecological, physiological and demographic contexts in which social behaviour is expressed. By modifying habitat structure, climatic conditions, resource dynamics and sensory or chemical environments, human activity indirectly alters movement patterns, encounter rates, energetic constraints and risk landscapes, thereby changing the costs and benefits of social association. In this section, we examine how such indirect effects of human activity affect sociability across taxa, focusing on habitat modification, climate‐driven processes, and pollution as key pathways through which social interactions and social structure are reorganised without direct human–animal contact.

#### 4.1.1 Habitat Modification

The expansion of human populations is accompanied by widespread modification of natural landscapes, fundamentally altering the spatial context in which social interactions occur. Because physical proximity is a prerequisite for social contact in most animals, changes in habitat structure—such as openness, complexity and the presence of barriers—can strongly influence individuals' opportunities to associate with conspecifics by shaping movement patterns and encounter rates (He et al. 2019).

Open habitats often promote increased grouping, either by elevating predation risk or by removing physical constraints on movement and social contact. In fish, for example, individuals form larger and more cohesive groups in structurally simple environments, particularly under predation pressure (Orpwood et al. 2008; Webster et al. 2013). Conversely, structural complexity may also increase social connectivity by funnelling individuals into shared pathways or habitat patches. In sleepy lizards (*Tiliqua rugosa*), experimentally increased habitat complexity led to greater social connectivity and stability, likely by constraining movement and increasing repeated encounters among individuals (Leu et al. 2016). These contrasting outcomes highlight that habitat modification can either promote or inhibit social associations depending on how it reshapes movement and encounter probabilities.

Acute or recurrent disturbance can further reorganise social interactions by altering spatial associations or imposing costs of disturbance avoidance. Fire disturbance in tree lizards ( *Urosaurus ornatus* ) altered spatial association networks, shifting individuals' positions towards geographically closer neighbours and altering patterns of individual connectivity at more frequently disturbed sites which probably affected the dynamics of their interactions (Lattanzio and Miles 2014). In other cases, avoiding anthropogenic disturbance may itself impose time or energetic constraints on social behaviour. For example, grey wolves ( *Canis lupus* ) exposed to increasing human modification of the landscape exhibited reduced pair cohesion, suggesting that disturbance avoidance can indirectly erode social bonds (Benson et al. 2025).

Habitat modification can also affect social behaviour by altering risk landscapes and resource distributions. Forest conversion for agriculture, which constitutes the main reason behind the global loss of forest habitats, not only fragments habitats but also exposes animals to novel threats and anthropogenic food sources (FAO 2020; Holzner et al. 2021). In southern pig‐tailed macaques, individuals ranging within the interior of oil palm plantations showed reduced rates of affiliative interactions and increased aggression. At plantation edges close to forest habitat, however, grooming rates and social partner diversity were higher than in the forest, while social network structure also differed, with low‐ranking rather than high‐ranking individuals occupying the most central positions (Holzner et al. 2021). These patterns suggest that increased visibility and perceived risk, rather than food availability alone, can constrain social tolerance and reshape affiliative behaviour and social networks in modified habitats.

Landscape change can also arise when animals are introduced into novel environments, where social interactions may facilitate behavioural adjustment through social learning. Reintroduced woolly monkeys ( *Lagothrix lagotricha* ) adapted more rapidly to new habitats when groups included experienced conspecifics, highlighting the role of social partners in mediating responses to habitat novelty (Gómez‐Muñoz et al. 2024). However, social preferences do not always align with maximising learning opportunities. Translocated elephants initially preferentially associated with other translocated individuals rather than local residents, forming socially segregated networks that gradually dissolved as the environment became familiar and the ratio of costs to potential benefits of such associations diminished (Pinter‐Wollman et al. 2009).

Together, these examples illustrate that habitat modification alters the ecological conditions under which individual social tendencies are expressed. By reshaping movement constraints, risk landscapes and resource distributions, it may change both individual patterns of association and the extent of expressed sociability, with consequences for emergent social structure. Individuals must therefore balance the benefits of social association against its costs in modified environments—trade‐offs that are not yet fully understood for many species and that may lead to apparently maladaptive social decisions under novel conditions.

#### 4.1.2 Climate Change

Climate change alters the social environment by reshaping energetic constraints, demographic stability, and the spatial and temporal distribution of resources. Through these pathways, rising temperatures, increasing climatic variability and extreme weather events modify the balance of costs and benefits associated with expressed sociability. As a result, climate‐driven environmental change can affect not only the frequency of social interactions but also the structure, cohesion, and functional consequences of social networks. Below, we synthesise evidence for how climate change influences expressed sociability via (i) temperature‐related physiological constraints, (ii) climate‐driven changes in resource availability, and (iii) catastrophic events that impose abrupt disruptions to social organisation.

##### 4.1.2.1 Temperature Rise

Rising temperatures can alter the balance of costs and benefits associated with expressed sociability by modifying physiological constraints on individuals. In cold environments, aggregation often functions as a mechanism of thermal buffering, reducing heat loss and energetic expenditure. As temperatures increase, these benefits may diminish, leading to reduced grouping and altered social dynamics.

In endotherms and ectotherms alike, aggregation can lower individual thermoregulatory costs, particularly for small‐bodied animals with high surface area‐to‐volume ratios. For example, common bushtits ( *Psaltriparus minimus* ) form dense huddles during cold periods, but cease clumping once temperatures rise (Smith 1972). Similarly, desert night lizards ( *Xantusia vigilis* ) form social aggregations that enhance thermal buffering and improve juvenile survival. Modelling suggests that rising temperatures may substantially reduce the frequency of such beneficial aggregations in the future (Rabosky et al. 2012). In these systems, warming is expected to decrease expressed sociability by relaxing physiological constraints that previously favoured close social association.

In warmer environments, elevated temperatures can instead increase the costs of social interaction by reshaping time budgets and activity patterns. Individuals may be forced to allocate more time to inactivity, seek cooler microhabitats or engage in active thermoregulation, thereby reducing opportunities for social contact. In sociable weavers ( *Philetairus socius* ), high and variable temperatures increase panting behaviour and are associated with less cohesive and more fragmented social networks (Rat et al. 2020). Such reductions in network cohesion may have downstream consequences for cooperation and information flow within groups.

In ectotherms, temperature directly affects metabolic rates and oxygen demand, further modifying the social environment. Higher temperatures can elevate energetic requirements, intensify competition and increase disease transmission risk, potentially tipping the balance such that the costs of sociability outweigh its benefits. Consistent with this, sticklebacks originating from allopatric warmer habitats exhibit lower sociability than those from colder environments. Furthermore, it was found that sociability levels are not only heritable but also influenced by rearing temperature (Pilakouta et al. 2023). Moreover, sociability was repeatable across trials in individuals acclimated to low temperatures but not in those exposed to warm conditions, indicating that temperature can influence the stability and expression of interindividual differences in social behaviour across contexts (Pilakouta et al. 2023).

In aquatic systems, warming‐induced reductions in dissolved oxygen further interact with temperature effects on expressed sociability. Hypoxic conditions have been shown to disrupt schooling behaviour in fish, which is often used as a proxy for sociability in these animals, leading to increased spacing among individuals and altered group structure (Domenici et al. 2017). Although multiple climate‐related stressors often co‐occur, their combined effects on expressed sociability remain poorly understood, highlighting an important gap in current research (Tiddy, Munson, et al. 2024).

Together, these findings indicate that rising temperatures can reshape expressed sociability through both direct physiological effects and indirect constraints on activity and interaction. Rather than producing uniform behavioural responses, warming alters the social landscape in ways that depend on species ecology and thermal history, with effects that can scale from individual sociability to network cohesion and group structure.

##### 4.1.2.2 Resource Scarcity

Climate‐driven changes in temperature and precipitation regimes frequently translate into altered resource availability, thereby reshaping the balance between the costs and benefits of sociality. Because expressed sociability mediates access to food, information and cooperative opportunities, shifts in resource distribution can profoundly affect social relationships and grouping patterns.

Reduced or spatially heterogeneous resource availability is generally expected to intensify competition, which may constrain affiliative behaviour and favour more dispersed social strategies. However, under certain ecological conditions, sociality can mitigate the costs of scarcity. Cooperative foraging, information sharing, and coordinated movement may allow individuals to exploit limited or patchily distributed resources more efficiently.

Experimental work in European shore crabs ( *Carcinus maenas* ) illustrates this context dependence. When preferred resources were clumped, individuals with low exploratory tendencies formed cohesive, stable subgroups, and thus achieved foraging success comparable to that of more exploratory, solitary individuals, likely through movement synchronisation and access to social information (Tanner and Jackson 2012). When preferred resources were dispersed, no stable aggregations were observed, highlighting how expressed sociability interacts with resource distribution to shape foraging strategies.

Indeed, when environmental conditions reduce the need for cooperation or increase the costs of social association, expressed sociability may decline. In common ravens ( *Corvus corax* ), cooperative alliances facilitate access to carcasses during periods of food scarcity, particularly among juveniles competing with dominant adults (Heinrich and Marzluff 1995). These alliances are supported by social associations within roosts that function as information centres. When food becomes more readily available, however, the benefits of such social ties diminish, potentially favouring more independent strategies.

Resource‐driven shifts in expressed sociability are particularly evident in social carnivores. In grey wolves, pair cohesion varies with prey size and availability: cohesion decreases when prey are small and competition within pairs increases, whereas larger prey promote greater cooperation (Benson et al. 2025). Pair cohesion was also lower under greater climatic variability, suggesting that both resource conditions and broader environmental variability can shape the stability of social associations.

Comparable patterns emerge in other taxa, where environmentally driven changes in individual association patterns scale up to reorganise social networks. In African lions ( *Panthera leo* ), social network structure within prides varies with prey abundance, size and spatial distribution, with stronger subdivision into subgroups occurring when prey are abundant and dispersed, and more uniform association patterns when prey are scarce or aggregated (Mbizah et al. 2020). Spotted hyenas ( *Crocuta crocuta* ) similarly increase social association rates during periods of low rainfall, potentially as a strategy to locate unevenly distributed prey (Ilany et al. 2015).

In contrast, prolonged or anthropogenically intensified resource shortages may substantially reduce the expression of sociability. Following forest and peat fires in Borneo, extended fruit scarcity reduced association rates among orangutan females and their weaned offspring, as well as among related and unrelated adult females, while increasing rates of agonistic interactions between unrelated females (Ashbury et al. 2022). Although social patterns eventually returned to pre‐disturbance states, these findings show that sustained resource scarcity can temporarily shift individual social behaviour away from affiliation and towards avoidance or conflict.

In species characterised by fission–fusion dynamics, resource scarcity often manifests as changes in subgroup size and stability. After hurricanes in Mexico, spider monkeys ( *Ateles geoffroyi yucatanensis* ) formed smaller subgroups and in the consecutive dry season exhibited fewer fusions, likely reflecting constraints imposed by reduced food quality and availability (Schaffner et al. 2012). Similarly, in southern resident killer whales ( *Orcinus orca* ), years of low Chinook salmon abundance were associated with reduced group sizes and less interconnected social networks, whereas high prey availability promoted greater gregariousness and network cohesion (Foster et al. 2012).

Taken together, these examples demonstrate that resource scarcity does not uniformly increase or decrease expressed sociability. Instead, it reshapes social behaviour by altering the relative payoffs of cooperation, competition and information sharing. Importantly, anthropogenically driven resource fluctuations differ from historical patterns in their duration, spatial scale and predictability, potentially pushing social systems beyond the range of conditions under which they evolved. As a result, changes in expressed sociability under resource scarcity may have cascading consequences for population stability, information flow, and resilience to further environmental perturbations.

In many systems, these resource‐mediated effects are further amplified when scarcity results from extreme climatic disturbances.

##### 4.1.2.3 Catastrophic Events

Climate change is associated with increasing frequency and intensity of extreme weather events, such as hurricanes, cyclones and large‐scale fires, which can rapidly disrupt habitats and population structure (Ummenhofer and Meehl 2017; van de Pol et al. 2017). These events often result in abrupt losses of resources and individuals, forcing survivors to adjust their behaviour under novel and highly unstable conditions. Unlike gradual climatic shifts, catastrophic events can push social systems beyond the range of conditions under which behavioural plasticity alone is sufficient to maintain stability.

In the immediate aftermath of catastrophic events, animals frequently exhibit increases in expressed sociability that may function as a compensatory response to heightened environmental uncertainty. Following hurricanes, rhesus macaques increased the number of affiliative social connections, particularly among individuals that were previously socially isolated (Testard et al. 2021). Rather than strengthening existing bonds, individuals expanded their social networks, potentially enhancing access to social support and tolerance during a period of heightened stress. Similar short‐term increases in social associations have been observed in other taxa, including Atlantic spotted dolphins ( *Stenella frontalis* ) and red‐backed fairy wrens ( *Malurus melanocephalus* ), where disturbance‐driven displacement led to increased contact rates as individuals congregated in less‐affected habitat patches (Elliser and Herzing 2014; Lantz and Karubian 2017).

Despite these short‐term benefits, increased expressed sociability following catastrophic events can entail delayed and system‐level costs. In the same rhesus macaque population, simulations incorporating post‐hurricane social networks revealed that pathogen infection risk doubled, up to 5 years after the event, compared with pre‐hurricane conditions (Motes‐Rodrigo et al. 2023). Moreover, infection risk became more evenly distributed across social ranks, as formerly low‐ranking individuals increased their number of social partners and approached the connectivity of higher‐ranking group members. These findings highlight how short‐term social buffering may be offset by longer‐term epidemiological consequences.

The social consequences of catastrophic events may extend beyond immediate population declines, disrupting both group organisation and patterns of social interaction. In black howler monkeys ( *Alouatta pigra* ), hurricanes caused severe population declines and a period of social disorganisation characterised by small, unstable groups and frequent solitary individuals (Pavelka et al. 2003). Although social structure gradually re‐emerged, overall rates of social interaction remained lower than before the disturbance, likely reflecting altered demographic composition, reduced juvenile presence and the formation of groups composed of previously unfamiliar individuals (Behie and Pavelka 2005).

Together, these examples illustrate that catastrophic climatic events can profoundly reshape expressed sociability and social structure, but the direction and persistence of these changes vary across systems. While increased expressed sociability may enhance short‐term resilience by promoting social support and information exchange, it can also generate delayed costs through altered disease dynamics or prolonged social instability. More broadly, indirect anthropogenic changes reshape expressed sociability by altering the ecological and demographic contexts in which social decisions are made, thereby modifying the costs, benefits, and functional consequences of social interactions without necessarily involving direct contact between animals and humans. Understanding these trade‐offs is essential for predicting population responses to increasingly frequent extreme events under ongoing climate change.

#### 4.1.3 Pollution

Anthropogenic pollutants can affect social behaviour not only through lethal toxicity but also via sublethal physiological, neurological and sensory stress, with consequences that are often expressed in the social domain. Aquatic organisms are particularly vulnerable to such effects, as pollutants readily accumulate in water bodies and directly interfere with metabolic and sensory processes (Tiddy, Cortese, et al. 2024). Behaviours fundamental to social organisation, such as shoaling and schooling, may therefore be altered even at pollutant concentrations that do not cause immediate mortality.

A meta‐analysis by Tiddy, Cortese, et al. (2024) demonstrated that exposure to organic and inorganic chemical pollutants, as well as artificial light, consistently reduces individual sociability in fish, while group‐level cohesion often remains unchanged. Notably, exposure to mixtures of chemical pollutants produced a contrasting pattern, increasing individual sociability but simultaneously reducing group cohesion (Tiddy, Cortese, et al. 2024). These findings highlight that pollution can decouple individual social tendencies from emergent group‐level properties, altering social structure in ways that may not be predictable from individual behaviour alone.

Pharmaceutical pollutants constitute a distinct category of chemical contamination, as many compounds are designed to act directly on neurological pathways that regulate behaviour. Exposure to such substances can therefore lead to pronounced but context‐dependent changes in expressed sociability. For example, European perch exposed to the anxiolytic oxazepam showed reduced shoaling behaviour (Brodin et al. 2013). In contrast, exposure to fluoxetine increased shoaling propensity in guppies, but only under specific social conditions, such as male–female pairings (Mason et al. 2021). Together, these studies show that pharmaceutical pollutants can alter expressed sociability in different directions, with the magnitude of these effects depending on the compound, exposure conditions and social context.

Beyond chemical contamination, sensory pollution—particularly anthropogenic noise—has emerged as an important driver of social behavioural change. In several bird species, experimentally elevated traffic noise increased rates of close social association and reduced nearest‐neighbour distances, leading to tighter group clustering (Owens et al. 2012; Hawkins et al. 2024). Such responses may function as antipredatory strategies, as noise pollution impairs the detection of predator cues and elevates perceived risk, making increased social proximity advantageous.

However, noise pollution can also disrupt social behaviour by degrading communication signals essential for maintaining social networks. In red‐backed fairy wrens, chronic exposure to traffic noise reduced social connectivity, likely due to interference with vocal communication that mediates between‐group interactions (Hawkins et al. 2020). This contrast illustrates that sensory pollution can either increase or decrease expressed sociability depending on whether its dominant effect is heightened perceived risk or impaired information transfer.

Together, these findings indicate that pollution can reshape expressed sociability at the individual level and broader patterns of social organisation through multiple pathways, including physiological stress, neuromodulation, and sensory disruption. Importantly, pollutant effects may differ between individual‐level social tendencies and group‐level cohesion, underscoring the context‐dependence across levels of social organisation. Such divergence across levels may have cascading consequences for information flow, cooperation, and population resilience in polluted environments.

### 4.2 Direct Consequences of Human Activity

Direct anthropogenic effects on expressed sociability arise when animals are exposed to humans as immediate ecological and social agents. Direct human presence, interactions and exploitation confront animals with novel sources of disturbance, risk, and opportunity that must be incorporated into social decision‐making in real time. These effects operate through multiple pathways, including altered stress levels, time budgets, perceived predation risk and access to anthropogenic resources. As a result, direct human activity can reshape social behaviour at the level of individuals, reorganise social networks within groups and destabilise population‐level social structure. In this section, we synthesise evidence for how direct interactions with humans—from everyday coexistence to lethal removal—modify expressed sociability and its consequences across taxa.

#### 4.2.1 Human Presence and Interactions With Humans

The expansion of human populations has not only transformed landscapes but has also increased the direct presence of humans in habitats previously occupied exclusively by wildlife. For many species, humans now constitute a novel and persistent component of the social environment, altering animals' behaviour not only through habitat modification but through direct disturbance, risk and interaction. Human presence can therefore reshape expressed sociability by modifying stress levels, time allocation, perceived predation risk and access to resources.

Direct human presence may suppress affiliative social behaviour by increasing vigilance and reducing opportunities for social interaction. In California ground squirrels ( *Otospermophilus beecheyi* ), individuals habituated to humans and their dogs showed reduced affiliative interactions and spent less time on social foraging, in contrast to responses elicited by natural predators, whose presence rather promoted social cohesion (Gall et al. 2022). Similarly, spotted hyena clans inhabiting areas with high human activity exhibited less dense social networks, suggesting reduced interaction rates among group members (Belton et al. 2018). In giraffes, proximity to human settlements was associated with weaker and more exclusive social relationships among females, indicating disruption of social structure rather than simple avoidance (Bond et al. 2021). These examples suggest that humans can function as a distinct class of disturbance, qualitatively different from natural predators, with unique consequences for social behaviour.

At the same time, human presence is a potent source of stress, raising the possibility that increased expressed sociability may function as a coping mechanism. According to the social buffering hypothesis, affiliative social interactions can mitigate the physiological and behavioural consequences of stress. Evidence from long‐tailed macaques ( *Macaca fascicularis* ) supports this view: individuals living in landscapes with higher human impact exhibited more grooming and more diverse grooming networks, potentially using social affiliation to cope with heightened stress (Marty et al. 2019). However, stress‐related behaviours were not always directly linked to rates of human–animal interactions, making it difficult to attribute increased grooming specifically to human‐induced stress.

These stress‐mediated responses are counterbalanced by time constraints imposed by human presence. Animals are limited in the number of behaviours they can perform simultaneously, and increased vigilance or monitoring of humans may reduce time available for social interactions. In urban and semi‐urban primates, including rhesus and bonnet macaques ( *Macaca radiata* ), individuals that spent more time monitoring human activity groomed less, had fewer social partners, and engaged in shorter and less reciprocal grooming bouts (Kaburu et al. 2018, 2019; Balasubramaniam et al. 2020). These findings support the time‐constraint hypothesis, whereby interactions with humans indirectly suppress expressed sociability by reallocating time towards vigilance and risk assessment.

Access to anthropogenic food sources introduces an additional layer of complexity. Energy‐rich human food can reduce foraging time and potentially free time for social interaction, consistent with the free‐time hypothesis. In semi‐provisioned Assamese macaques ( *Macaca assamensis* ), individuals from groups relying more heavily on human food spent less time feeding and more time engaging in social behaviour than conspecifics from wild‐feeding groups (Koirala et al. 2017). However, these effects depend critically on the nature of human–animal interactions. In vervet monkeys, increased expressed sociability was observed only when anthropogenic food access involved benign provisioning, whereas conflictual interactions with humans reduced social behaviour (Thatcher et al. 2019). By contrast, chimpanzee party cohesion increased during crop‐raiding—when risk from humans was high—but not when feeding on abandoned food, suggesting that perceived danger can promote social cohesion in anthropogenic contexts (Hockings et al. 2012).

Within populations, different forms of human‐associated foraging can produce divergent social outcomes. In bottlenose dolphins, individuals engaging in direct begging from humans showed lower social centrality following a harmful algal bloom, whereas dolphins foraging around fixed fishing gear exhibited higher centrality and larger group sizes (Bankhead et al. 2025). At a broader scale, changes in fisheries practices altered dolphin social networks, with reduced trawling enabling previously segregated communities to merge into a more connected network (Ansmann et al. 2012). These findings underscore that direct human activities can both fragment and reconnect social systems, depending on how they modify interaction opportunities and risk landscapes.

Together, these studies demonstrate that direct human presence does not exert uniform effects on expressed sociability. Instead, it introduces novel trade‐offs between stress, time, risk, and resource access, leading to context‐dependent reorganisation of social behaviour and social networks. While many of these changes may be flexible and reversible, sustained human pressure can set the stage for more profound and lasting social disruption, particularly when direct interactions escalate into lethal removal or population decline, as discussed in the following section.

#### 4.2.2 Poaching and Loss

Direct human‐caused mortality and removal can alter expressed sociability and social structure not only by reducing population size but also by disrupting social relationships and the network architecture through which information, cooperation, and social support are maintained. Classic work on the Allee effect suggests that the fitness of individuals and the growth of populations can decline at low densities, making undercrowding particularly costly for social species (Allee 1931). While habitat fragmentation (Section 4.1.1) primarily constrains expressed sociability by reducing encounter rates and spatial connectivity, direct human‐caused loss alters expressed sociability through the removal of social partners and disruption of established social roles. In such cases, social disruption can act as a key mechanism linking mortality to reduced population resilience.

Evidence from the critically endangered Vancouver Island marmot ( *Marmota vancouverensis* ) illustrates how population decline can trigger a cascading breakdown of social behaviour and activity patterns, contributing to reduced survival and reproduction. Following steep declines associated with habitat alteration, contemporary marmots lived in smaller and more isolated colonies, exhibited markedly reduced affiliative interactions and greeting rates, and engaged more frequently in agonistic behaviour compared to historical populations (Brashares et al. 2010). These social changes were accompanied by increased ranging behaviour and antipredator vigilance, reduced feeding rates, and higher exposure to predation risk, together limiting individual fitness and constraining population recovery (Brashares et al. 2010). Recent work on captive Vancouver Island marmots further suggests that social stability and familiarity can influence stress physiology and reproductive success, underscoring the importance of social context for recovery in small populations (Graham et al. 2024).

Beyond changes in group size, the loss of social partners can trigger reorganisation of social networks among survivors. In some systems, individuals increase expressed sociability and form new connections following loss. Experimental removal of individuals from great tit social networks produced a positive relationship between flockmate loss and subsequent increases in association number, bond strength, and network connectedness (Firth et al. 2017). However, responses vary across species and may involve different strategies of compensation. In Canadian geese ( *Branta canadensis* ), culling did not lead to a strong increase in network connectivity. Instead, surviving individuals primarily strengthened existing relationships and formed new ties mainly with socially nearby individuals (Downing et al. 2023). Individual differences further shape these responses: in wild house mice ( *Mus musculus domesticus* ), less sociable individuals formed more new weak connections after large predation events, whereas more sociable individuals strengthened fewer bonds (Evans et al. 2020). Following sudden losses in eastern grey kangaroo mobs, pairwise associations increased, suggesting reduced selectivity and/or attempts to re‐establish social protection under heightened predation risk (Carter et al. 2009).

Loss of close associates can also induce strong stress responses, potentially promoting increased affiliative behaviour consistent with social buffering. In female chacma baboons ( *Papio ursinus* ), the death of close relatives was followed by increased grooming rates and a broader grooming network, coinciding with the reduction back to baseline of the elevated glucocorticoid levels, suggesting that affiliative contact may mitigate stress after bereavement (Engh et al. 2006).

Importantly, human‐caused mortality is often non‐random and may disproportionately remove individuals occupying key social roles. Network analyses of northeastern Pacific resident killer whales showed that social networks were robust to random removals but highly sensitive to targeted removals biased towards socially central juvenile females, mimicking historical live captures, which produced fragmentation into isolated components (Williams and Lusseau 2006). Simulated targeted poaching of socially central individuals in African savanna elephants reduced population‐level network connectivity, indicating that the removal of highly connected individuals can degrade social structure even when fragmentation does not occur (Wiśniewska et al. 2022). Similarly, in bottlenose dolphins, temporary disappearance of ‘broker’ individuals with high betweenness reduced interactions between communities without fully collapsing network structure, illustrating that social cohesion can erode even when networks appear superficially intact (Lusseau and Newman 2004). More generally, removals of individuals performing stabilising functions can have cascading social effects, as illustrated above by the experimental removal of policing individuals in pig‐tailed macaques (Flack et al. 2006).

For cooperative species, direct human‐caused mortality can destabilise social organisation even if overall abundance does not immediately decline. In wolves, pack structure underpins cooperative hunting, territory defence and reproductive success. Human‐caused mortality can trigger pack dissolution and reduce reproduction through disruption of leadership and social cohesion (Cassidy et al. 2023). In elephants, poaching often targets older individuals that function as social hubs and repositories of ecological knowledge (McComb et al. 2011; Goldenberg et al. 2016). The loss of such individuals can weaken social connectivity and disrupt knowledge transmission that is particularly valuable under environmental stress, such as low resource availability (McComb et al. 2001; Brent et al. 2015).

Finally, repeated exposure to poaching, culling, translocation and social fragmentation can produce long‐lasting behavioural and neurophysiological effects consistent with trauma. In elephants, social disruption has been linked to impaired social competence and altered decision‐making, particularly in groups founded by orphaned individuals following culls (Bradshaw et al. 2005; Shannon et al. 2013). At the same time, social support may buffer some of these consequences: in mountain gorillas, orphans have increased social integration and bond strength, which may contribute to maintaining survival despite maternal loss (Morrison et al. 2021).

Together, these studies show again that direct human‐driven loss can reshape expressed sociability through multiple pathways—from reduced density and partner loss to targeted removal of socially central individuals and long‐term trauma—with consequences that extend from individual behaviour to population‐level resilience.

### 4.3 Synthesis: Common Mechanisms and Context Dependence

Across the diverse examples reviewed above, anthropogenic environmental change reshapes expressed sociability through a limited set of recurrent mechanisms, while the direction and magnitude of responses remain strongly context dependent. At a fundamental level, human‐driven change alters the cost–benefit landscape of social association by modifying energetic constraints, predation risk, disease exposure, and access to information and resources. Increased environmental uncertainty—arising from climatic variability, disturbance, or unpredictable human activity—often amplifies the value of social buffering and information sharing, favouring increased association or broader social networks. At the same time, changes in social network position, particularly the roles of highly connected or central individuals, repeatedly emerge as key determinants of population‐level consequences, from information flow to disease transmission. A recurring trade‐off is evident: expressed sociability can provide short‐term buffering against stress and uncertainty, yet generate delayed costs through heightened competition, epidemiological risk, or network destabilisation.

These shared mechanisms do not produce uniform outcomes. Crucially, responses at different levels need not align: changes in individual social behaviour may leave group cohesion largely unchanged, whereas shifts involving only a subset of socially influential individuals can reorganise networks and generate population‐level consequences. The direction and magnitude of responses are further shaped by species ecology, social system organisation, individual phenotype and social role, and the duration, predictability, and spatial distribution of anthropogenic disturbance. Short‐term, acute disturbances may favour transient increases in social association, whereas prolonged or unpredictable pressures can erode social tolerance and destabilise social structure. Recognising both the common mechanistic pathways and their context‐dependent expression is essential for interpreting apparently contradictory findings and for understanding how social systems respond to accelerating anthropogenic change.

## 5 Implications

In the context of accelerating anthropogenic change and ongoing biodiversity loss, sociability emerges as a trait with direct relevance for conservation biology and wildlife management. Because individual variation in sociability shapes social networks, information flow and patterns of contact among individuals, it can either hinder or facilitate conservation interventions. Ignoring social structure and individual social tendencies risks undermining efforts aimed at disease control, conflict mitigation, population recovery and species translocation.

### 5.1 Sociability, Social Networks and Disease Management

Infectious diseases pose a major threat to endangered wildlife populations and can also mediate zoonotic and zooanthroponotic spillover to humans. Because pathogen transmission depends on patterns of contact among individuals, both the intensity and structure of social connections are critical for understanding outbreak dynamics (Cantor et al. 2021). Social network analysis has therefore become an important tool for identifying individuals that are highly connected or occupy bridging positions within networks, as such individuals may disproportionately contribute to pathogen spread (Krause et al. 2010).

However, the relationship between sociability, network position, and disease risk is not straightforward. In Tasmanian devils ( *Sarcophilus harrisii* ), Hamede et al. (2009) showed that targeted removal of highly connected individuals was of limited effectiveness for controlling devil facial tumour disease, because individual network positions changed seasonally and were not stable targets for intervention. By contrast, simulation studies in wild chimpanzees demonstrated that vaccination strategies targeting the most connected individuals could prevent large outbreaks with substantially fewer vaccines than random vaccination (Rushmore et al. 2014). These contrasting outcomes highlight that the utility of network‐informed disease management depends on the stability of social roles and the repeatability of individual sociability.

In human‐dominated landscapes, disease dynamics are further complicated by interactions with people. Model analyses based on data from peri‐urban macaque populations revealed that both grooming networks and co‐interaction networks involving humans shaped zooanthroponotic outbreak size, with more centrally positioned individuals driving larger outbreaks (Balasubramaniam et al. 2022). Importantly, outbreak size was not predicted by the overall frequency of human–macaque interactions, but by the structure of social connections around humans. These findings underscore that sociability, expressed in specific social contexts, can critically influence disease risk even when mean interaction rates appear unchanged.

Together, these studies demonstrate that sociability mediates disease dynamics through its effects on social network structure, but that management strategies must account for temporal variability, social plasticity and the specific pathways of transmission involved.

### 5.2 Sociability, Social Learning and Human–Wildlife Conflict

Socially transmitted behaviour can exacerbate human–wildlife conflict when animals learn to exploit anthropogenic resources. In such cases, understanding how information spreads through social networks is essential for designing effective management strategies. Network‐based diffusion simulations have shown, for example, that culling programmes aimed at limiting socially transmitted foraging behaviour in California sea lions ( *Zalophus californianus* ) would have been most effective if implemented early, before social transmission amplified recruitment into the behaviour (Schakner et al. 2016). Delayed intervention allowed socially connected individuals to facilitate rapid spread, increasing both ecological impact and the number of animals ultimately killed.

This example illustrates how social transmission can spread a foraging strategy that benefits individual animals while intensifying conservation conflict and generating longer‐term risks for the population. From a management perspective, this implies that interventions targeting behaviour, rather than individuals per se, must be timed and designed with an explicit understanding of social transmission pathways.

### 5.3 Sociability in Conservation Interventions and Population Management

Individual differences in sociability can also influence invasion dynamics, translocation success, and responses to conservation interventions. In invasive Trinidadian guppies ( *Poecilia reticulata* ), within‐population variation in sociability towards conspecifics and heterospecifics may facilitate invasion by allowing individuals to integrate into native shoals when conspecific density is low (Camacho‐Cervantes and Ojanguren 2024). Individuals without strong association preferences may promote early establishment, while those more sociable towards conspecifics may later reinforce group cohesion and invasion success.

Expressed sociability is also relevant for conservation translocations and reintroductions, where animals are exposed to novel environments and elevated stress. Social buffering—the mitigation of stress through affiliative contact—has been documented even in species not typically considered social. In southern Pacific rattlesnakes ( *Crotalus helleri* ), the presence of a companion reduced physiological stress responses following disturbance (Martin, Fox, et al. 2023). Such findings suggest that considering expressed sociability and social context may improve translocation outcomes and animal welfare.

Finally, expressed sociability can shape responses to research and management practices themselves. Reactions to novel objects, such as drones or tracking devices, may vary with group size and social context. In guanacos ( *Lama guanicoe* ), individuals in larger groups, assumed more sociable, showed stronger avoidance responses to drones than solitary animals or those in smaller groups (Schroeder and Panebianco 2021). Such patterns indicate that both expressed sociability and social organisation can modulate behavioural responses to disturbance and should be considered when designing monitoring protocols.

### 5.4 Synthesis

Across these applied contexts, sociability provides an individual‐level lens linking behavioural variation to population‐level outcomes (Figure 2). By facilitating disease spread, accelerating social learning, buffering stress, or shaping responses to intervention, both interindividual differences in sociability and context‐dependent changes in its expression can influence conservation outcomes. Incorporating these dimensions of sociability, together with social network structure, into management frameworks therefore offers a promising, though still underutilised, avenue for conservation in increasingly human‐dominated environments.

**FIGURE 2:**
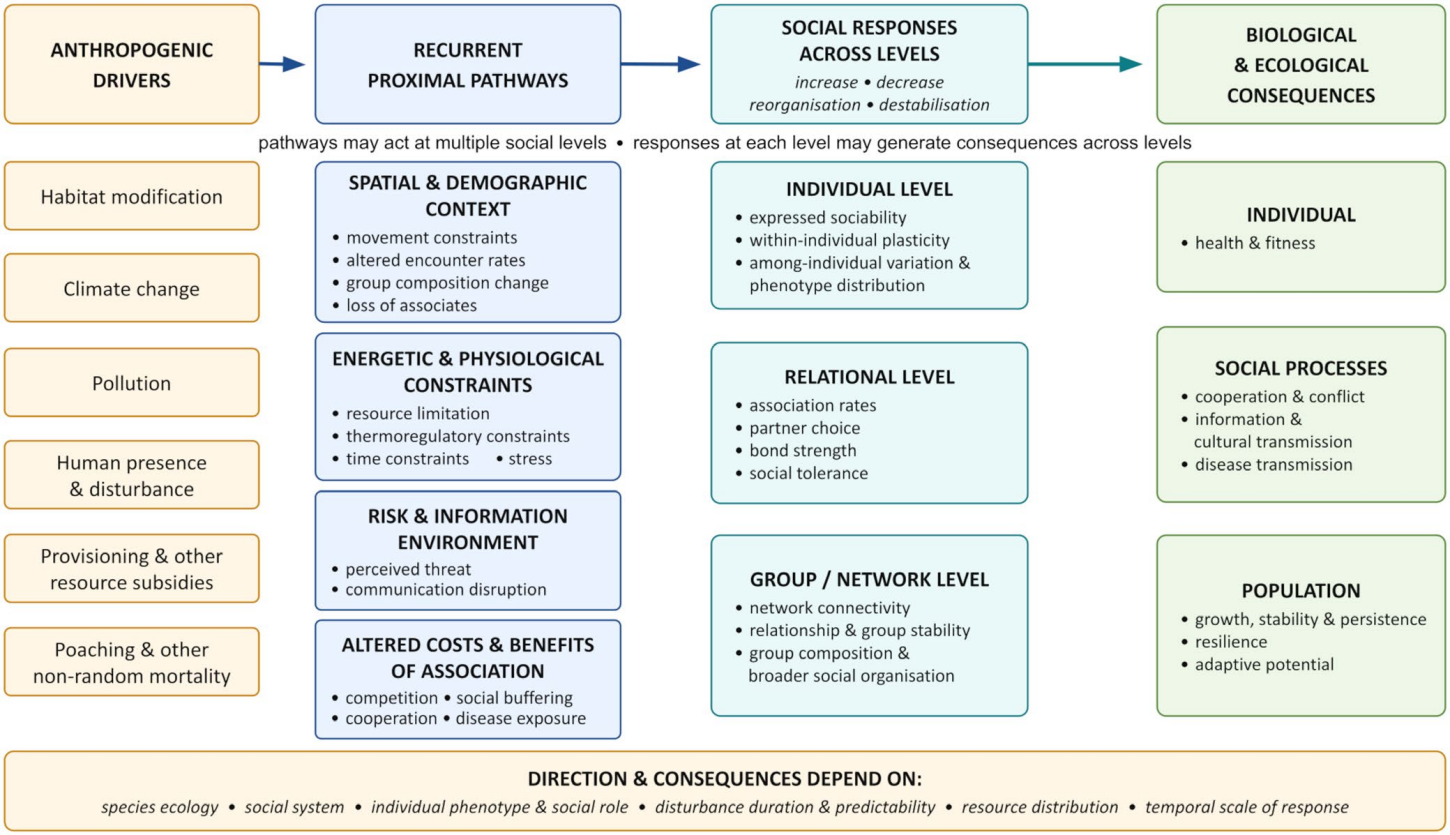
Conceptual synthesis of the recurrent proximal pathways through which anthropogenic change reshapes social behaviour across individual, relational and group/network levels and generates biological and ecological consequences. Pathways may act simultaneously across levels, and responses may involve increases, decreases, reorganisation or destabilisation. Their direction and consequences depend on ecological, social, individual and temporal context. Changes in expressed social behaviour do not necessarily imply changes in sociability as a repeatable personality trait.

## 6 Conclusions

1. Anthropogenic change reshapes the cost–benefit landscape of social associations through multiple pathways.
2. Observed responses are strongly context dependent and lack a generalisable direction; however some recurrent mechanisms can be distinguished often operating simultaneously across multiple levels of social organisation and having broad ecological consequences.
3. Individual‐level responses can scale up through social relationships and networks to reshape population‐level processes, while changes in the social environment feed back on individual behaviour and fitness.
4. Short‐term increases in the expressed sociability can buffer disturbance but generate delayed or system‐level costs.
5. Many documented social responses involve shifts in expressed social behaviour or social network structure and do not, by themselves, demonstrate changes in sociability as a personality trait.
6. Social disruption can have persistent and delayed consequences, including developmental effects that only emerge over long timescales.
7. Preserving variation in individual social phenotypes and in social relationships may be critical for resilience under rapid environmental change.

## Author Contributions

**Paulina Kowalczyk:** conceptualization (equal), writing – original draft (lead), writing – review and editing (supporting). **Barbara Pietrzak:** conceptualization (equal), writing – review and editing (lead).

## Funding

The authors have nothing to report.

## Conflicts of Interest

The authors declare no conflicts of interest.

## Data Availability

The authors have nothing to report.
